# Perspectives of Non-Medical Professionals Working in a Mental Health Service on Eating Disorders: Awareness, Knowledge, and Stigmatisation

**DOI:** 10.3390/nu17243878

**Published:** 2025-12-12

**Authors:** Hakan Öğütlü, Uğur Tekeoğlu, Fiona McNicholas

**Affiliations:** 1 Department of Child & Adolescent Psychiatry, Cognitive Behavioral Psychotherapies Association, Ankara 06680, Turkey; hogutlu@gmail.com; 2 Department of Child & Adolescent Psychiatry, Faculty of Medicine, Recep Tayyip Erdoğan University, Rize 53100, Turkey; 3 Department of Child & Adolescent Psychiatry, School of Medicine and Medical Science, University College Dublin, D04 V1W8 Dublin, Ireland; fiona.mcnicholas@sjog.ie

**Keywords:** eating disorders, non-medical professionals, awareness, stigma, training, Türkiye

## Abstract

**Background**: Eating disorders (EDs) are serious mental health conditions often beginning in adolescence and are associated with significant morbidity and mortality. Although previous research has focused on medical staff, little is known about how EDs are recognised and managed by non-medical mental health professionals within community-based systems. This study examined their awareness, knowledge, and stigmatising beliefs toward EDs in Türkiye, highlighting implications for training and policy development. **Methods**: A study-specific questionnaire adapted from a validated tool previously used with general practitioners was administered to 42 non-medical professionals (95.2% female; mean age = 33.2 ± 5.6 years) working in community mental health services in Ankara, Türkiye. Participants were randomly assigned to one of six clinical vignettes depicting a young person with anorexia nervosa (AN), bulimia nervosa, binge eating disorder, avoidant/restrictive food intake disorder (ARFID), depression, or type-1 diabetes. The questionnaire assessed illness perception, causal attributions, emotional reactions, professional knowledge, and stigmatising beliefs. **Results**: Only 28.6% (*n* = 12) correctly identified the disorder in their assigned vignette. BED had the highest diagnostic accuracy (50%), while AN and ARFID were most frequently misidentified. Participants commonly believed that EDs mainly affected females, had a short duration, and were less responsive to treatment. Stigmatising beliefs—such as personal blame—were also noted. **Conclusions**: Findings revealed limited awareness and knowledge of EDs—particularly AN and ARFID—among non-medical mental health professionals. Misconceptions that EDs are self-inflicted may delay recognition and referral. The results underscore the need for targeted education, anti-stigma interventions, and integration of ED content into professional training to improve early detection and community-based support.

## 1. Introduction

Eating disorders (EDs) are psychiatric conditions associated with substantial morbidity, mortality, and healthcare burden. In Türkiye, the point prevalence of EDs is estimated to be 2.3%, with a gender distribution of approximately 4.0% in females and 0.6% in males, consistent with rates observed in other Western countries [1]. In addition, increases in eating disorder symptoms and the severity of these symptoms have been reported during and after the pandemic [2,3].

The development of EDs depends on numerous variables ranging from sociocultural, biological, and genetic to psychological factors [4,5]. EDs are serious, relatively chronic conditions associated with comorbid psychopathology and adverse medical conditions. Individuals with EDs are at higher risk of suicide attempts and death than both the general population and individuals with other psychiatric disorders as well as complex physical complications [6,7]. The mortality rate in patients with anorexia nervosa (AN) is the highest among psychiatric conditions, with high suicide rates and deaths from physiological causes [6]. In addition, the cost of AN treatment is higher than schizophrenia [8].

The impairment in functioning caused by EDs may be comparable in severity to anxiety and depression [9]. All EDs have significant comorbidity with many other psychiatric disorders. Although relatively rare, EDs are an important public health problem as they are often associated with other psychopathology and personality disorders and are frequently undertreated [10].

The medical complications of EDs are significant, potentially irreversible, and life-threatening. Careful patient explanation of the medical changes that may occur when a patient has an eating disorder can help patients make positive changes [11]. Various behaviours and characteristics, such as eating disorder-related behaviours and comorbidity, have been found to affect quality of life. As the symptom severity of the eating disorder increases, quality of life deteriorates more, and it has been shown that quality of life is affected even in subclinical or subthreshold cases [12,13].

Findings from previous studies suggest that people with EDs face significant delays in accessing appropriate treatment [14]. Most do not seek treatment, and among those who do, the tendency is to seek help with diet and weight loss, or for comorbid mental or physical illness. One study reported a substantial unmet need for treatment, with only 17–31% of individuals with EDs in the community seeking disorder-specific care [15]. On average, there is a delay of more than five years between the onset of symptoms and the initiation of appropriate treatment [14]. Several barriers contribute to this treatment gap including insufficient knowledge among health professionals, the normalisation of disordered eating behaviours, stigma, and limited access to specialised care [16].

Lack of awareness means that EDs are often not recognised by clinicians [7]. Even among clinicians, knowledge of EDs is low. Studies conducted in the USA and Ireland reported low levels of knowledge and confidence in ED treatment among medical service providers (including general practitioners, paediatricians, and nurses) [17,18]. Even psychiatrists, who are expected to provide specialised services, reported dissatisfaction with the training they received [19]. In addition to specific knowledge gaps, health professionals surveyed in the Irish study exhibited more negative attitudes towards EDs than other mental or physical illnesses, and stated that they preferred to work with individuals with depression rather than those with EDs [20]. There are also encouraging findings: a study of school counsellors investigating knowledge and awareness of EDs found a reasonable level of knowledge and few stigmatising attitudes [21].

Another factor causing delayed diagnosis is stigmatisation. Stigma has been reported as one of the most influential barriers to treatment seeking for all EDs [14,22]. It has important but often neglected implications for effective care. Weight biases held by therapists can be particularly harmful for patients and hinder treatment progress [23]. ED-related stigma may lead to psychological consequences such as low self-esteem and feelings of shame and guilt [24,25]. It has also been shown to exacerbate the course of illness: more frequent experiences of ED-related stigma are associated with higher levels of ED psychopathology [26], while stigma-induced alienation and social withdrawal predict more severe symptoms [27]. Stigma has further been linked to longer illness duration and reduced treatment seeking [24,26,28].

Currently, the vast majority of individuals worldwide who need mental health services for EDs and other psychiatric problems do not receive treatment [29]. Although health service utilisation among those with EDs is increasing, many remain undiagnosed or untreated despite contact with health professionals [15]. For example, about two-thirds of primary care physicians reported being unable to obtain outpatient mental health services for their patients, a rate at least twice that observed for other health needs [30]. Lack of mental health service providers has also been identified as a significant barrier to access, and this challenge varies depending on the clinician’s practice, health system structure, and policy factors [18].

There is also evidence that EDs are common in outpatient mental health patients but often remain hidden and unrecognised [22]. Given these challenges, there is a widespread need for training to enhance the recognition of symptoms and appropriate referral pathways [15]. In particular, it is vital that issues regarding awareness are incorporated into training programs for professionals.

In the Turkish health system, non-medical mental health professionals such as social workers, psychologists, counsellors, child development specialists, and teachers do not directly diagnose EDs but play a crucial role in early recognition, screening, psychoeducation, family guidance, and referral to specialised care. Their responsibilities are especially important given the scarcity of specialised clinicians, as they often serve as the first point of contact for individuals with subclinical symptoms or families seeking advice.

Beyond the Turkish context, international studies highlight similar gaps in awareness among non-medical providers, underscoring the importance of cross-country comparisons and knowledge exchange. Recent innovations, such as digital platforms and artificial intelligence-based tools for personalised ED treatment, have also emerged [31], demonstrating the need to prepare non-medical professionals to collaborate within evolving, technology-supported models of care.

Given these gaps, this study aimed to investigate the recognition, identification, knowledge, and attitudes of non-medical professionals working in mental health services toward EDs in Türkiye. Specifically, it examined their diagnostic accuracy using vignettes, perceived causal attributions, stigmatising attitudes, and the influence of demographic and professional factors.

## 2. Materials and Methods

### 2.1. Study Design

This study employed a cross-sectional, vignette-based survey design to evaluate the awareness, knowledge, and stigmatising beliefs of non-medical professionals towards eating disorders (EDs). Participants were randomly assigned to one of six clinical vignettes depicting the following conditions: AN (*n* = 8), bulimia nervosa (BN) (*n* = 7), binge eating disorder (BED) (*n* = 8), avoidant/restrictive food intake disorder (ARFID) (*n* = 7), type 1 diabetes (*n* = 7), and depression (*n* = 5). The inclusion of depression and T1DM vignettes enabled comparisons between EDs and other psychiatric/medical conditions, thereby assessing diagnostic accuracy, perceived causal factors, and stigma in a broader context. Randomisation was conducted automatically using a computer-generated sequence embedded in the Qualtrics platform to ensure unbiased allocation.

### 2.2. Participants

Ethical approval for this study was obtained from the Ankara City Hospital Clinical Research Ethics Committee (Approval No: E1-20-258, Date: 13 February 2020). The recruitment process was conducted as follows. A comprehensive list of all non-medical professionals working in the mental health services of the Ankara Metropolitan Municipality and the Ankara Provincial Health Directorate was obtained, comprising 50 eligible individuals. All 50 potential participants were approached in person at their respective workplaces by a member of the research team. They were provided with detailed information about the study’s aims, procedures, confidentiality, and their right to withdraw. Of the 50 individuals approached, 49 agreed to participate and provided written informed consent in a face-to-face setting, yielding a response rate of 98%. Data were successfully collected from 42 of these participants. The high response rate significantly minimises the potential for selection bias, as the final sample represents the vast majority (84%) of the entire accessible target population in these key Ankara-based institutions.

Inclusion criteria were: (i) being a non-medical professional working in a mental health setting, (ii) currently practicing in Ankara, Türkiye, and (iii) providing informed consent. The exclusion criteria were defined as incomplete survey responses, being a medical doctor, and not providing informed consent. One participant whose professional role was undefined was excluded from the final analyses to maintain data validity. Participants included psychologists, social workers, child development specialists, and nurses specifically employed in mental health units.

Information was collected regarding the participants’ prior training and education in the field of EDs. It was noted that most participants had very limited or no formal training in this area. We acknowledge that the relatively small sample size (*n* = 42) may limit the generalizability of these findings, and this limitation is addressed in the Discussion.

### 2.3. Materials and Procedures

A study-specific questionnaire (SSQ), previously used in a study of general practitioners in Ireland, was adapted for this research [20]. Cronbach’s alpha values of the SSQ subscales have been reported to range from 0.76 to 0.89 [20]. The SSQ was translated from English to Turkish and then back to English by a professional translator. The original and back-translated versions were compared for vocabulary, meaning, and content, and necessary modifications were made. The SSQ includes the Illness Perception Questionnaire and five case vignettes depicting a young person with AN, BN, BED, depression or type 1 DM. An additional vignette and two related questions on ARFID were added and piloted with a group of non-medical mental health professionals. With the SSQ, participants were assigned only one vignette, and the vignette was randomly selected.

Participants were asked to describe the disease in the vignette, the causes of the disease, the patient’s affective reaction, the patient’s personality traits, their feelings about interacting with the patient, and their beliefs about stigma in the community. The SSQ also collected the participants’ body mass index (BMI), body satisfaction, and sociodemographic information. The participants’ professional knowledge of EDs was also assessed with the SSQ.

#### 2.3.1. Diagnosis and Cause of the Disease

(1) Diagnosis: With open-ended items adapted from Mond and Hay [32], participants were asked to indicate, after reading a randomly assigned vignette, what the ‘main problem’ of the young person in the vignette was and what steps they would usually take to diagnose the problem. Participants were asked to identify the vignettes as one of the diagnostic options AN, BN, BED, ARFID, DM, and depression. In addition, the participants’ answers were classified by the researchers as belonging to one of the four diagnostic groups (EDs, DM, depression, and other problems).
(2) Cause of the disease: Participants indicated their beliefs about the potential causes of the disease (overwork, family problems, stress or worry, emotional state, personal behaviour, mental attitude, personality, diet or eating habits, chance or bad luck, poor medical care, and hereditary factors). Responses were rated on a 5-point Likert scale (1 = strongly disagree, 3 = neutral, 5 = strongly agree) with the SSQ.

#### 2.3.2. Attitudes Towards the Target

(1) Perceptions of illness: This was assessed using the adapted 5-point, 12-item version of the Illness Perceptions Questionnaire [33]. Participants’ beliefs about the likely timeline of the illness, the amount of personal control the individual has over the illness, and the effectiveness of treatment were assessed. Cronbach’s α scores were 0.58 for the timeline scale, 0.62 for personal control, and 0.61 for treatment [20].
(2) Affective reaction: Participants were asked to rate the emotional reactions of the character in the vignette for 10 different emotions, 5 positive (optimistic, supportive, empathic, comfortable, relaxed) and 5 negative (anxious, fearful, disgusted, nervous, irritable). Responses were rated on a 5-point Likert scale (1 = strongly disagree, 3 = neutral, 5 = strongly agree) with the SSQ.
(3) Personality traits: Participants were asked to rate their views on the personality traits of the vignette character for 10 different personality traits including 5 positive (strong, sociable, kind, intelligent, open) and 5 negative (insensitive, emotional, awkward, insecure, cold). Responses were rated on a 5-point Likert scale (1 = strongly disagree, 3 = neutral, 5 = strongly agree) with the SSQ.
(4) Emotions related to the interaction: Participants were asked to indicate their views on the typical reactions of mental health professionals when interacting with a patient like the one in the vignette (e.g., “I think mental health professionals generally find patients like the one in the vignette difficult to deal with”). Responses were rated on a 5-point Likert scale (1 = strongly disagree, 3 = neutral, 5 = strongly agree) with the SSQ.
(5) Gender of the target character: A gender-neutral Turkish name, Deniz, was used in each vignette. Participants were asked to assign a gender to the character ‘Deniz’ based on their reading of the vignette.

#### 2.3.3. Professional Knowledge of Eating Disorder

Since the participants were mostly professionals with administrative duties in governmental organisations, only professional knowledge of EDs was assessed with the SSQ; depression and DM were not included. Participants answered eight multiple-choice questions assessing their knowledge about the diagnosis and treatment of EDs including one diagnostic question and one treatment question for each of AN, BN, BED, and ARFID.

#### 2.3.4. Stigmatisation in the Community

(1) Social distancing: Participants were asked to rate the social marginalisation experienced by individuals with EDs (strain on friendship, reluctance for intimate relationships, employer reluctance to hire, reluctance to share housing, and unwillingness to be friends) using five items. Responses were rated on a 5-point Likert scale (1 = never, 3 = sometimes, 5 = always) with the SSQ.
(2) Stigmatising beliefs: Participants were asked to rate their agreement with five statements reflecting common stigmatising beliefs about individuals with EDs (individuals are fragile, responsible for their condition, would improve if they ate normally, can pull themselves together, use the disorder to get attention). Responses were rated on a 5-point Likert scale (1 = strongly disagree, 3 = neutral, 5 = strongly agree) with the SSQ.

### 2.4. Statistical Analysis

The data were analysed using IBM Statistical Package for Social Sciences (SPSS) version 17.0 for Windows (IBM Corp., Chicago, IL, USA, 2008). Normality was assessed using the Shapiro–Wilk test. Differences between two groups were analysed using the Student’s *t*-test and the Mann–Whitney U test. Differences among more than two groups were assessed using ANOVA and Kruskal–Wallis analysis. Post hoc analyses (Tukey and Tamhane’s T2 tests) were performed to compare multiple groups, and Bonferroni correction was applied. Pearson’s chi-square and Fisher’s exact tests were used to compare categorical variables. A *p*-value of <0.05 was considered statistically significant for comparisons. This study was conducted on a sample that encompassed almost the entire accessible population of non-medical mental health professionals in Ankara (*n* = 42/50, 84% participation rate). As the primary aim of the study was to provide qualitative insights in this underexplored area rather than conduct quantitative hypothesis testing, an a priori power analysis was not performed.

## 3. Results

### 3.1. Participant Demographics

Of the 42 participants, 95.2% were female (*n* = 40) and 4.8% were male (*n* = 2). The mean age of the participants was 33.2 ± 5.6 years (range: 24–45 years). Of the participants, 78.6% (*n* = 33) were employees of the Ankara Metropolitan Municipality, and 21.4% (*n* = 9) were employees of the Ankara Provincial Health Directorate. The mean duration of employment was 8.3 ± 4.6 years (range: 1–19 years). The professional composition of the participants included 42.9% (*n* = 18) social workers, 2.4% (*n* = 1) counsellors, 19% (*n* = 8) child development specialists, 14.3% (*n* = 6) teachers, 16.7% (*n* = 7) psychologists, 2.4% (*n* = 1) nurses, and 2.4% (*n* = 1) undefined. One participant whose role was “undefined” was excluded from further analyses to minimise bias.

### 3.2. Diagnosis and Cause of the Disease

**Figure 1:**
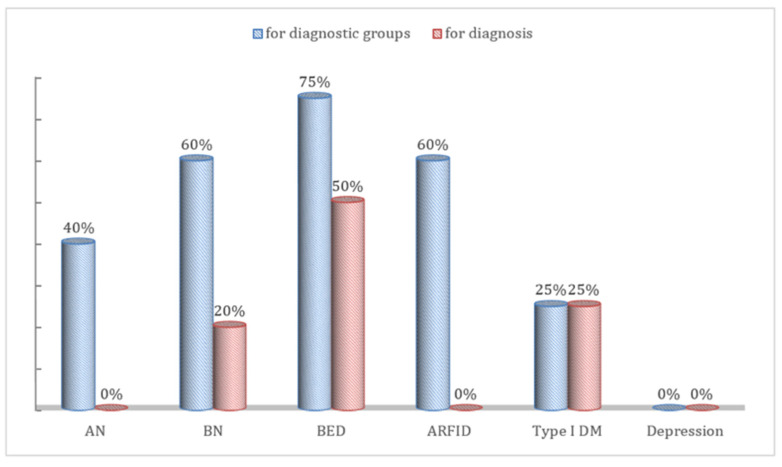
Percentages of correct diagnosis for each vignette (correct diagnosis rates were determined for a diagnosis and one of four diagnostic groups (EDs, DM, depression, other problems). “Diagnosis” refers to the raw responses provided by the participants, while “diagnostic group” refers to the classification of these responses by the researchers.

Of the 42 participants, 19.0% (*n* = 8) read a vignette with a diagnosis of AN, 16.7% (*n* = 7) BN, 19.0% (*n* = 8) BED, 16.7% (*n* = 7) ARFID, 16.7% (*n* = 7) Type 1 DM, and 11.9% (*n* = 5) major depressive disorder. Only 28.6% (*n* = 12) of participants correctly identified the vignette diagnosis, with the highest accuracy observed for BED cases (50%). Detailed percentages for all diagnostic groups are presented in Figure 1. The highest referral rate was to a psychiatrist (*n* = 18, 42.9%), followed by a psychologist (*n* = 17, 40.5%). Only one participant (2.4%) suggested referring the patient to a dietician.

Participants’ attributions of illness causes, as assessed by the SSQ, did not differ significantly across diagnostic groups (*p* > 0.05). Family problems and mental attitude were slightly more often cited for EDs than for depression or diabetes, though the differences were not significant (see Table 1).

### 3.3. Attitudes

Significant group differences were found for the illness perception timeline (*p* = 0.040) and negative personality features (*p* = 0.038). Post hoc analyses showed that the timeline difference occurred between participants who perceived the vignette as depression and those who viewed it as other issues (*p* = 0.036). For negative personality features, participants who identified the vignette as depression tended to rate the character more negatively, though pairwise comparisons did not remain significant after correction.

Other attitudes—including treatment efficacy, illness duration, and personal control—did not differ significantly across diagnostic groups (*p* > 0.05). Participants generally perceived treatment for EDs and depression as less effective and viewed patients with EDs as having shorter illness duration and lower personal control.

No significant group differences were found in affective reactions, perceived personality traits, or feelings about interacting with the vignette character. However, fewer positive personality traits were attributed to ED cases than to other diagnostic groups. Detailed comparisons are shown in Table 2.

### 3.4. Target Gender

Participants were asked to indicate their assumptions about the gender of ‘Deniz’. The distribution of disorder groups by gender was similar (*p* = 0.275, Fisher’s exact test). Participants estimated that 64.3% (*n* = 9) of the cases they considered as EDs were female, while 35.7% (*n* = 5) were male.

### 3.5. Professional Knowledge of Eating Disorder

Participants’ professional knowledge about the diagnosis and treatment of EDs was higher for both BN (mean = 0.95 ± 0.69) and BED (mean = 4.44 ± 1.23) compared with AN (mean = 0.64 ± 0.61). ARFID had the lowest knowledge score among the EDs (mean = 0.47 ± 0.55).

### 3.6. Stigmatisation

Participants were asked to indicate Deniz’s status in the community regarding social distance and stigmatising beliefs. There was no significant difference (*p* > 0.05) between the diagnostic groups in terms of the degree of social marginalisation experienced in friendship, accommodation, or job opportunities compared with their peers without a diagnosis. There was also no significant difference (*p* > 0.05) in the extent to which participants perceived patients to be ‘fragile’, linked to an unhealthy diet, capable of ‘pulling themselves together’, or using their illness to attract attention. Participants held patients with EDs more responsible for their illness compared with other diagnostic groups, although this was not statistically significant. Stigmatising attitudes and beliefs based on the assigned diagnostic groups are shown in Table 3.

### 3.7. Body Satisfaction and Body Mass Index

Participants were asked to indicate their BMI and rate their degree of satisfaction with their weight and body shape using a Likert scale from 1 (very satisfied) to 5 (not at all satisfied). The mean weight of the participants was 66.5 ± 10.4 kg (range: 45–90 kg), mean BMI was 25.2 ± 4.9 (kg/m^2^) and the mean body satisfaction was 2.43 ± 0.94 (range: 1–5 points). There was no significant difference between body satisfaction and diagnoses (*p* = 0.796) and BMI and diagnoses (*p* = 0.713).

## 4. Discussion

The present study primarily aimed to assess the awareness, knowledge, and stigmatising beliefs of non-medical mental health professionals regarding eating disorders. The findings revealed that only 28.6% of participants were able to correctly diagnose EDs, highlighting a substantial gap in recognition. Professional knowledge about EDs was generally low, with AN and ARFID being the least accurately identified. Moreover, participants tended to attribute family problems as a causal factor for EDs, perceived ED treatment as less effective than for other conditions, and more frequently assigned female gender to vignette cases with EDs. These findings underscore the urgent need for improved training and awareness among non-medical professionals.

Participants in this study tended to misestimate the types of EDs, possibly due to a lack of awareness of the different aspects of these disorders. It is crucial for mental health professionals to understand the differences between EDs in various respects [34]. The findings of this study suggest that instead of viewing EDs as a whole, it is important to consider their subtypes.

The results of this study concern the inadequate knowledge of non-medical mental health professionals about EDs. Only 28.6% of the participants were able to correctly diagnose EDs, likely due to their lack of awareness and knowledge on the subject. Previous studies have shown that even psychiatrists’ knowledge of EDs varies due to specific gaps in both diagnosis and treatment [17,19]. Considering this, it is expected that non-medical mental health professionals diagnose EDs less accurately. It is also important to note that the primary responsibility of non-medical professionals may not be to provide formal diagnosis, but rather to screen, recognise risk, raise awareness in the community, and ensure the timely referral of suspected cases to specialist services. Their supportive roles can still play a crucial part in early detection and intervention.

AN and ARFID were the least correctly diagnosed EDs. The level of professional knowledge about EDs was directly related to the ability to correctly diagnose these disorders. AN is a serious eating disorder with a high suicide rate, a chronic course in about 20% of cases, and an almost 18-fold increase in mortality, with more than half of the patients having another psychiatric disorder [6]. While this finding for AN may come as unexpected, previous studies suggest that primary care providers and medical professionals, including general practitioners and nurses, have inadequate knowledge about AN, which contributes to misdiagnosis and under-referrals [17,19]. The lack of professional knowledge and awareness among all health workers about a highly fatal disease such as AN is a critical issue that needs to be addressed.

The lowest level of professional knowledge was observed for ARFID. This finding for ARFID is consistent with other studies. Although ARFID is not a new disorder, it was not clearly defined and characterised until the publication of the DSM-5 in 2013. ARFID involves persistent and clinically significant impairment in meeting nutritional and/or energy intake requirements without body image disturbances [35]. The diagnosis of ARFID according to the DSM-5 requires significant weight loss or stunted growth, nutritional deficiencies, dependence on supplementary feeding, and/or psychosocial impairment [36]. Reasons for food avoidance or restriction include a lack of interest in food, sensory characteristics of food, fear of choking, or emotional problems [37]. Studies have shown that ARFID negatively impacts health-related quality of life [38]. Individuals with ARFID may experience serious medical consequences such as amenorrhea, weight loss, low bone mineral density, electrolyte imbalances, bradycardia, and heart problems [39,40].

The prevalence of ARFID in the general adolescent population is similar to that of AN and BED [41]. A systematic review reported that the estimated prevalence of ARFID in a non-medical sample of children and adolescents ranged from 0.3% to 15.5% [42]. Patients with ARFID often present initially to non-psychiatric settings, making its assessment and treatment important for all healthcare professionals [43]. However, little is known about the clinical features of ARFID in non-medical samples [44].

Individuals with ARFID frequently seek help from various health practitioners who may not specialise in mental health treatment and are unfamiliar with ARFID. One study found that clinicians reported a lack of training in ARFID assessment and were not familiar with its diagnostic criteria [45]. Given the prevalence of ARFID and its associated physical, nutritional, and psychosocial burdens, both clinicians and non-medical health professionals must be competent in managing the needs of people with ARFID [38,41]. The present findings reinforce the gap in ARFID knowledge and highlight this disorder as a priority target for future training initiatives.

Although the study participants did not report statistically significant differences between any etiologic cause for the conditions presented to them, they tended to cite familial problems as the cause of EDs more often than other diagnostic groups. Empirical evidence, particularly in adolescent EDs, confirms the importance of familial relationships in the development and maintenance of pathology [46]. Families of female adolescents with EDs are characterised by problematic profiles in family functioning including interpersonal boundary issues, poor conflict tolerance, and low overall family satisfaction [47]. Overdependence on family members, low flexibility, poor communication, and overprotectiveness are other findings in these families [48]. Lack of necessary support during transitional family events may also accelerate the onset of EDs or contribute to the persistence of the illness [49]. Future interventions should include family-based treatment approaches that have proven efficacy in the treatment of adolescent EDs [50].

Participants tended to perceive treatment effectiveness for EDs as lower than for other diagnostic groups, even when no significant difference was observed. This perception may negatively impact patient referrals for treatment. Previous research has shown that health professionals’ pessimistic attitudes towards ED treatment can reduce the likelihood of early intervention and appropriate referrals [26]. However, studies indicate that early intervention significantly improves the recovery rates, underscoring the need to shift professional attitudes through targeted training and awareness campaigns [29]. It is possible that the participants’ responses were influenced by the specific content of the vignettes provided, as case descriptions may shape illness perceptions and expectations about treatment outcomes.

The burden of caregiving for individuals with mental illness can be significant and increases the urgency of early referral to specialist services [51]. Caregiving burden has been found to be higher for carers of patients with EDs than for those caring for patients with depression or schizophrenia [52]. Despite this, access to effective treatments for EDs remains limited [53]. Even with early and appropriate referral, patients with EDs may avoid seeking help. Barriers to help-seeking include stigma, shame, denial, failure to recognise the seriousness of the illness, treatment cost, low motivation to change, negative attitudes towards seeking help, lack of encouragement, and lack of information about sources of help [54]. Prevention and early intervention programs for EDs should focus on reducing stigma and shame, educating individuals about the seriousness of EDs, and increasing knowledge about help-seeking.

In clinical vignettes, non-medical mental health professionals were more likely to assign a female gender to the EDs depicted, aligning with epidemiological studies showing higher prevalence rates among girls [55]. Participants also considered male gender in cases presenting with eating problems, consistent with reports of increased prevalence and presentation of EDs in males [56]. Academic studies suggest that males account for approximately 25% of eating disorder cases in the community, but clinical cases are much lower (10% or less) [57]. This predominance of EDs in females may lead to overlooking male patients, delaying the recognition and treatment of symptoms, which can have serious consequences. Increasing the awareness of EDs in males is essential for ensuring equitable access to treatment and reducing disparities in care.

According to the results of this study, social stigmatisation in EDs is not different from depression and Type 1 DM. The fact that social stigmatisation towards patients with EDs did not differ significantly may be due to the effect of mental health campaigns to raise the awareness of stigmatisation. However, there was a tendency among participants to attribute personal responsibility for their illness, particularly to individuals with EDs, although this was not statistically significant. Similar tendencies have been noted in other studies, where EDs are often perceived as self-affecting conditions rather than serious psychiatric conditions. A large population-based study found that more than one-third of British adults believed individuals with EDs “should only blame themselves” and “should be able to pick themselves up” [58]. Similarly, an Australian study found that 30.3% of adults agreed somewhat or fully that BN patients were personally responsible for their condition [59]. A study of medical and nursing staff found that 51.5% and 59.3% of AN and BN patients, respectively, believed that patients were personally responsible for their condition [60]. Supporting the result of our study, a previous study using a similar methodology showed that there was no significant difference in the levels of social stigma between EDs, depression and Type 1 DM. However, participants endorsed the perception that young people with BN used their illness to ‘get attention’ more than those with depression (F = 3.468, *p* = 0.027) (mean BN 3.7 ± 0.5, mean depression 1.8 ± 0.8, *p* = 0.028) [21].

Stigma has been identified as a significant barrier to seeking treatment due to individuals’ fear of being judged by health professionals [54]. To address this issue, anti-stigma interventions should focus on reframing EDs as complex, multifactorial disorders requiring specialised care, rather than as lifestyle choices or personal failings. Addressing the perception that EDs are trivial and self-inflicted should be a focus of anti-stigma interventions.

The study had certain limitations. It was conducted in a selected province in Türkiye, with a small sample size (*n* = 42) and data limited by its subjective nature and retrospective recall. Therefore, the findings cannot be generalised to other regions in Türkiye. In addition, the vignette-based envelope methodology may limit ecological validity, as responses could have been shaped by case descriptions rather than real-world clinical encounters. The study also did not include an intervention program to assess whether training could improve knowledge or reduce stigma. Furthermore, the potential relationships between the participants’ demographic characteristics (e.g., age, professional experience) and the study outcomes were not examined. Other limitations include the regional restriction of the sample, the use of self-report measures, and potential social desirability bias in the participants’ responses. Further studies addressing these limitations will contribute to the literature and the development of training programs. Future research should expand the sample size and explore the impact of structured training programs on improving awareness and reducing stigma among non-medical mental health professionals.

## 5. Conclusions

The findings of the study are generally thought-provoking. The most concerning result is that the awareness and knowledge of eating disorders among non-medical professionals were low, and these disorders were often overlooked. Importantly, participants were asked about their prior training and education in the field of EDs, and most reported very limited or no formal training—thus this finding is based on collected data rather than assumption. To reduce the significant burden of EDs, future research and the work of health professionals must focus on developing, implementing, and evaluating effective programs that increase the search for appropriate and timely treatment. This responsibility also lies with health services, government, and funding agencies.

Given the scarcity of clinical mental health professionals and the difficulty in accessing them, non-medical mental health professionals can play a vital role in supporting subclinical cases and making appropriate referrals when necessary. However, when the literature was examined, it was seen that there are not enough studies on the perspective of non-medical mental health professionals on EDs. Implementing training programs for non-medical mental health professionals and making information easily accessible can contribute to the solution of these problems. Future strategies should also consider innovative tools, including digital training platforms and AI-supported approaches for personalised learning and early screening, which may enhance the capacity of non-medical professionals to identify and respond to EDs.

## Figures and Tables

**Table 1:** Participants’ perceived causal factors by diagnostic groups.

				Statistics	
Measure	Vignette Issue	*n*	Mean (SD)	F Value	*p* Value
**Overwork**	Eating disorders	14	2.4 (1.0)	0.077	0.972
	Depression	6	2.3 (0.5)		
	Type 1 DM	1	2.0		
	Other issues	4	2.5 (1.2)		
	**Total**	**25**	**2.4 (0.9)**		
**Family problems**	Eating disorders	14	3.7 (0.7)	0.774	0.521
	Depression	6	3.1 (0.9)		
	Type 1 DM	1	3.0		
	Other issues	5	3.6 (0.8)		
	**Total**	**26**	**3.5 (0.8)**		
**Stress or worry**	Eating disorders	14	4.0 (0.4)	6.971	0.002
	Depression	6	4.3 (0.5)	EDs vs. Depr.	NS
	Type 1 DM	1	2.0	EDs vs. others	NS
	Other issues	5	4.4 (0.5)	Depr. vs. others	NS
	**Total**	**26**	**4.1 (0.6)**	There are only one data for DM	NA
**Deniz’s emotional state**	Eating disorders	13	3.3 (1.3)	1.052	0.390
	Depression	6	4.3 (0.5)		
	Type 1 DM	1	3.0		
	Other issues	5	3.6 (0.8)		
	**Total**	**25**	**3.6 (1.1)**		
**Deniz’s own behaviour**	Eating disorders	13	3.5 (0.9)	0.890	0.463
	Depression	6	3.3 (0.8)		
	Type 1 DM	1	4.0		
	Other issues	4	2.7 (0.9)		
	**Total**	**24**	**3.3 (0.9)**		
**Deniz’s mental attitude**	Eating disorders	14	3.9 (0.8)	2.747	0.069
	Depression	6	3.8 (1.1)		
	Type 1 DM	1	1.0		
	Other issues	4	3.7 (1.2)		
	**Total**	**25**	**3.7 (1.0)**		
**Deniz’s personality**	Eating disorders	13	3.0 (1.3)	0.336	0.799
	Depression	6	3.1 (0.7)		
	Type 1 DM	1	4.0		
	Other issues	5	2.8 (0.8)		
	**Total**	**25**	**3.0 (1.0)**		
**Diet or eating habits**	Eating disorders	14	3.7 (1.1)	1.791	0.178
	Depression	6	3.6 (0.8)		
	Type 1 DM	1	2.0		
	Other issues	5	4.4 (0.5)		
	**Total**	**26**	**3.7 (1.0)**		
**Change or bad luck**	Eating disorders	14	1.5 (0.6)	2.050	0.136
	Depression	6	2.1 (0.9)		
	Type 1 DM	1	1.0		
	Other issues	5	1.2 (0.4)		
	**Total**	**26**	**1.6 (0.7)**		
**Poor medical care in Deniz’s past**	Eating disorders	14	2.4 (0.8)	1.851	0.167
	Depression	6	2.3 (0.8)		
	Type 1 DM	1	1.0		
	Other issues	5	3.2 (1.3)		
	**Total**	**26**	**2.5 (0.9)**		
**Hereditary**	Eating disorders	14	2.6 (0.7)	2.759	0.068
	Depression	5	3.2 (0.8)		
	Type 1 DM	1	5.0		
	Other issues	5	2.4 (1.3)		
	**Total**	**25**	**2.8 (1.0)**		

NA: not applicable, NS: not significant, SD: standard deviation, EDs: eating disorders, DM: diabetes mellitus. Note. Likert scale coded as 1 = strongly disagree, 5 = strongly agree. For cells with expected counts <5 (e.g., *n* = 1), Fisher’s exact test was used.

**Table 2:** Comparison of attitude in the context of diagnosis groups.

					Statistics	
Measure		Vignette Issue	*n*	Mean (SD)	F Value	*p* Value
**Illness perception**	**Treatment**	Eating disorders	13	3.5 (0.2)	1.454	0.257
		Depression	6	3.6 (0.2)		
		Type 1 DM	1	4.0		
		Other issues	4	3.1 (0.9)		
		**Total**	**24**	**3.5 (0.4)**		
	**Timeline**	Eating disorders	14	2.8 (0.3)	3.302	0.040
		Depression	6	3.1 (0.5)	Depr. vs. others	0.036
		Type 1 DM	1	3.5	EDs vs. Depr.	NS
		Other issues	4	2.4 (0.3)	EDs vs. others	NS
		**Total**	**25**	**2.8 (0.4)**	There are only one data for DM	NA
	**Control**	Eating disorders	13	3.2 (0.5)	1.101	0.372
		Depression	6	3.4 (0.4)		
		Type 1 DM	1	3.7		
		Other issues	4	2.8 (0.9)		
		**Total**	**24**	**3.2 (0.5)**		
**Affective reaction**	**Positive**	Eating disorders	12	3.8 (0.5)	1.873	0.170
		Depression	5	4.4 (0.4)		
		Type 1 DM	1	4.6		
		Other issues	4	4.1 (0.4)		
		**Total**	**22**	**4.0 (0.5)**		
	**Negative**	Eating disorders	12	1.7 (0.5)	0.674	0.580
		Depression	5	1.6 (0.4)		
		Type 1 DM	1	1.0		
		Other issues	3	1.7 (0.7)		
		**Total**	**21**	**1.7 (0.5)**		
**Personality features**	**Positive**	Eating disorders	11	2.9 (0.3)	0.924	0.452
		Depression	4	3.1 (0.5)		
		Type 1 DM	1	3.6		
		Other issues	4	2.8 (0.7)		
		**Total**	**20**	**2.9 (0.4)**		
	**Negative**	Eating disorders	11	2.7 (0.4)	3.519	0.038
		Depression	5	3.2 (0.2)	EDs vs. Depr.	NS
		Type 1 DM	1	2.2	EDs vs. others	NS
		Other issues	4	2.8 (1.6)	Depr. vs. others	NS
		**Total**	**21**	**2.8 (0.4)**	There are only one data for DM	NA
**Feelings regarding interaction**		Eating disorders	14	2.6 (0.4)	0.023	0.995
		Depression	5	2.6 (0.4)		
		Type 1 DM	1	2.6		
		Other issues	5	2.7 (0.4)		
		**Total**	**25**	**2.6 (0.3)**		

NA: not applicable, NS: not significant, SD: standard deviation, EDs: eating disorders, DM: diabetes mellitus. Note. Likert scale coded as 1 = strongly disagree, 5 = strongly agree. Fisher’s exact test was applied where appropriate.

**Table 3:** Stigmatising attitudes and beliefs based on assigned diagnostic groups.

				Statistics	
Measure	Vignette Issue	*n*	Mean (SD)	F Value	*p* Value
**Strain on friendship**	Eating disorders	12	3.4 (0.5)	0.446	0.723
	Depression	6	3.1 (0.7)		
	Type 1 DM	1	4.0		
	Other issues	3	3.0 (2.0)		
	**Total**	**22**	**3.3 (0.8)**		
**Not want intimate relationship**	Eating disorders	12	2.8 (1.0)	0.659	0.588
	Depression	6	2.8 (0.7)		
	Type 1 DM	1	4.0		
	Other issues	3	2.3 (1.5)		
	**Total**	**22**	**2.8 (1.0)**		
**Employers reluctant to hire**	Eating disorders	11	3.1 (0.7)	1.045	0.374
	Depression	5	3.4 (1.3)		
	Type 1 DM	0	0		
	Other issues	3	2.3 (1.5)		
	**Total**	**19**	**3.1 (1.0)**		
**Reluctant to share house**	Eating disorders	11	3.0 (0.6)	2.827	0.070
	Depression	6	2.8 (0.9)		
	Type 1 DM	1	4.0		
	Other issues	3	1.6 (1.1)		
	**Total**	**21**	**2.8 (0.9)**		
**Others unwilling to be friends**	Eating disorders	12	3.0 (0.9)	2.021	0.147
	Depression	6	2.8 (0.4)		
	Type 1 DM	1	3.0		
	Other issues	3	1.6 (1.1)		
	**Total**	**22**	**2.8 (0.9)**		
**Deniz is fragile**	Eating disorders	12	3.3 (0.7)	2.262	0.114
	Depression	6	3.3 (0.5)		
	Type 1 DM	1	4.0		
	Other issues	4	2.2 (1.2)		
	**Total**	**23**	**3.1 (0.8)**		
**Deniz responsible for condition**	Eating disorders	12	3.3 (0.8)	0.686	0.571
	Depression	6	3.0 (0.6)		
	Type 1 DM	1	2.0		
	Other issues	4	3.2 (1.5)		
	**Total**	**23**	**3.1 (0.9)**		
**Deniz would get better if just ate normally**	Eating disorders	12	3.9 (0.6)	2.011	0.147
	Depression	6	3.1 (0.9)		
	Type 1 DM	1	4.0		
	Other issues	4	2.7 (1.5)		
	**Total**	**23**	**3.5 (0.9)**		
**Deniz able to pull self together**	Eating disorders	12	3.3 (0.8)	0.730	0.547
	Depression	6	3.3 (1.0)		
	Type 1 DM	1	5.0		
	Other issues	4	3.5 (1.7)		
	**Total**	**23**	**3.4 (1.0)**		
**Deniz uses disorder to get attention**	Eating disorders	12	2.7 (0.7)	0.910	0.455
	Depression	6	2.5 (0.8)		
	Type 1 DM	1	4.0		
	Other issues	4	2.5 (1.2)		
	**Total**	**23**	**2.6 (0.8)**		

SD: standard deviation, DM: diabetes mellitus. Note. For the first five items, the Likert scale was coded as 1 = strongly disagree, 5 = strongly agree, and for the second five items, a separate Likert scale was coded as 1 = never, 5 = always. Fisher’s exact test was applied where appropriate.

## Data Availability

The original data presented in the study are openly available in http://doi.org/10.6084/m9.figshare.29204609 (accessed on 28 October 2025).

## Abbreviations

The following abbreviations are used in this manuscript:

AN	Anorexia nervosa
ARFID	Avoidant/restrictive food intake disorder
BED	Binge eating disorder
BN	Bulimia nervosa
BMI	Body mass index
DM	Diabetes mellitus (Type 1)
DSM-5	Diagnostic and Statistical Manual of Mental Disorders, Fifth Edition
ED	Eating disorder
EDs	Eating disorders
IPQ-R	Revised Illness Perception Questionnaire
MDD	Major depressive disorder
SPSS	Statistical Package for the Social Sciences
SSQ	Study-Specific Questionnaire
